# Clarifying the Tooth-Derived Stem Cells Behavior in a 3D Biomimetic Scaffold for Bone Tissue Engineering Applications

**DOI:** 10.3389/fbioe.2020.00724

**Published:** 2020-06-26

**Authors:** Christiane L. Salgado, Cristina C. Barrias, Fernando J. M. Monteiro

**Affiliations:** ^1^i3S – Instituto de Investigação e Inovação em Saúde, Universidade do Porto, Porto, Portugal; ^2^INEB, Instituto Nacional de Engenharia Biomédica, Universidade do Porto, Porto, Portugal; ^3^ICBAS, Instituto de Ciências Biomédicas Abel Salazar, Universidade do Porto, Porto, Portugal; ^4^FEUP, Faculdade de Engenharia da Universidade do Porto, Departamento de Engenharia Metalúrgica e Materiais, Porto, Portugal

**Keywords:** dental pulp and follicle stem cells, 3D multicompartment holder, bone tissue engineering, biomaterials, collagen, nanohydroxyapatite

## Abstract

Massive amounts of cell are needed for creating tissue engineered 3D constructs, which often requires culture on scaffolds under dynamic conditions to facilitate nutrients and oxygen diffusion. Dynamic cultures are expected to improve cell viability and proliferation rate, when compared to static conditions. However, cells from distinct types and/or tissues sources may respond differently to external stimuli and be incompatible with culture under mechanical shear stress. The first aim of this work was to show that dental stem cells are a valuable source for improving bone regeneration potential of artificial grafts. Mesenchymal stem/stromal cells (MSCs) were isolated from human dental follicle (hDFMSC) and pulp tissues (hDPMSC) and shown to express prototypical stem cell markers. The follicle and pulp dental MSCs capacity to differentiate into osteoblast lineage was evaluated after seeding on 3D porous scaffolds of collagen-nanohydroxyapatite/phosphoserine biocomposite cryogel with osteogenic factors in the culture medium. Both tooth-derived MSCs were able to show high ALP activity, express osteogenic gene markers and secrete osteopontin (OPN). Thereafter, designed multicompartment holder adaptable to spinner flasks was used for dynamic culture (50 rpm) of both dental MSCs types within the porous 3D scaffolds. Standard static culture conditions were used as control. Culture under dynamic conditions promoted follicle MSCs proliferation, while improving their spatial distribution within the scaffold. Under dynamic conditions, the biocomposite scaffold promoted MSCs osteogenic differentiation, as suggested by increased alkaline phosphatase (ALP) activity, higher osteogenic gene expression and OPN deposition. In a similar manner, under dynamic conditions, dental pulp MSCs also showed higher ALP activity and proliferation rate, but lower amounts of osteopontin secretion, when compared to static conditions. After implantation, dental follicle MSCs-loaded 3D scaffolds cultured under dynamic conditions showed higher tissue ingrowth and osteogenic differentiation (higher human OPN secretion) than dental pulp cells. Overall, this study explored the use of tooth-derived stem cells as a clinical alternative source for bone tissue engineering, together with an innovative device for dynamic culture of cell-laden 3D scaffolds. Results showed that human MSCs response upon culture on 3D scaffolds, depends on the cells source and the culture regimen. This suggests that both the type of cells and their culture conditions should be carefully adjusted according to the final clinical application.

## Introduction

Maxillofacial large bone defects may derive from various causes, such as traffic accidents, falls, inflammatory process, oral tumors, and others, both on their own or in combination with other injuries (Lee, 2012). The surgical treatment relies on amputation of large portions of hard and soft tissue, generating major problems to patients, causing very limitative disabilities and significantly reduced quality of life. Clinical solutions for bone repair include allografts, autografts and commercially available bone grafts eventually associated to titanium internal fixation implants. Therapies employing autografts present the disadvantage of requiring a second surgical site to obtain the donor-bone, consequently increasing the risk of co-morbidity. On the other hand, natural and synthetic bone grafts need only one surgery, but repairing these bone tissues requires the reconstruction of their biological and functional properties, which cannot be accomplished yet through such clinical strategies (Amini et al., 2012).

In a Tissue Engineering strategy, biomimetic 3D scaffolds are a fundamental tool for bone regeneration, but cell adhesion and expansion on the material’s surface, fulfilling the overall structure is still a challenge yet to be tackled. Human mesenchymal cell source is a key part to achieve the promise of tissue regeneration. There is the need for high quality adult stem cells from an easily accessible source (Ratajczak et al., 2016). As alternative cell lines, isolated human dental pulp and follicle stem cells were confirmed to show multipotency, and self-renewal capability (Morsczeck et al., 2005), thus being a suitable alternative source of stem cells for the purpose of cell-based therapies for hard-tissue engineering such as for craniofacial defects, as well as in alveolar bone defects. They are also a suitable alternative source of stem cells for the purpose of cell-based therapies in regenerative medicine due to their multilineage differentiation potential as well as their immunomodulatory properties, where they should interplay to suppress excessive inflammation during tissue repair (Sharpe, 2016). The minimally invasive isolation of these stem cells from extracted third molars raised high hopes for potential clinical applications (Sharpe, 2016). Recently, dental pulp stem cells showed bone regeneration efficacy similar to the one of bone marrow MSCs *in vivo* and should be a promising cell source for bone defects repair (Lee et al., 2019). In addition, dental follicle stem cells were found to be the precursor cells of periodontal tissues cells (PTCs) including fibroblasts in the periodontal dental ligament (PDL), alveolar bone cells, and cementoblasts. In addition, studies have shown an abundance of bioactive molecules, factors, and proteins related to dental tissue formation in the mineralized matrix (Yao et al., 2008).

The cell response and stability when cultured inside 3D porous scaffolds in a tissue engineering (TE) assay should require 3D cell culture technologies. Dynamic culture has previously shown to positively affect human MSCs proliferation, differentiation, and ECM production when compared to static 3D cultures (Woloszyk et al., 2014). A dynamic culture system should improve nutrients and oxygen diffusion, avoiding hypoxia-induced central necrosis in cultured tissue constructs (Tsai et al., 2019). However, some bioreactors could promote hydrodynamic stress during cell culture resulting in shear stress, thus causing cell damage (Tanzeglock et al., 2009). Some types of dynamic conditions may also generate air bubbles, and bubble–liquid interphase has shown to cause cells damages (Walls et al., 2017). In this context, dynamic culture systems, such as spinner flasks, are widely used due to their ability to improve mass transfer in cell cultures suspension. However, few studies describe the use of spinner flasks for cell cultures in three-dimensional (3D) scaffolds, because this culture system type is often unsuitable, since several factors, as scaffold geometry, porosity and hydrophilicity should influence the cellular vitality and proliferation rate of cells seeded on/inside porous 3D scaffolds (Gelinsky et al., 2015). To address hypoxia-induced central necrosis in cultured tissue constructs, a multi-compartment holder adaptable to spinner flasks for 3D cell-loaded materials culture was developed in-house (Teixeira et al., 2014). The implementation of adequate dynamic conditions is critical to obtain successful 3D cultures and favor the use of 3D scaffolds as an approach closer to physiological conditions in tissue engineering (Barrias and Goncalves, 2013). This proposed device should protect matrices with low mechanical strength from the shear stress promoted by the stirring process, avoiding inappropriate floating exposure.

Numerous types of biocomposite materials based on collagen and nanohydroxyapatite are widely studied (Teixeira et al., 2010; Rodrigues et al., 2013; Salgado et al., 2016) and are well known to improve bone regeneration and fulfil small craniofacial defects. These composite materials could have surface modifications in order to improve cell behavior, favor mesenchymal stem cells adhesion and control cell differentiation into the desired cell types, allowing the regeneration of the host tissue/organ. Phosphoserine (O-phospho-L-serine) is a phosphorylated amino-acid (OPS) and shall be able to mimic typical osteopontin functionalities, such as the regulation of cell response, such as mitosis (proliferation), signaling, differentiation (D’Ambrosio et al., 2007), and also provide osteoconductive properties (Salgado et al., 2019).

The objectives of this work were to isolate tooth-derived stem cells (follicle and pulp tissues) as potential stem cell sources and evaluate their capacity to differentiate *in vitro* into bone-like cells in a Collagen-nanohydroxyapatite/OPS (Coll-nanoHA/OPS) biomimetic 3D scaffold. After that, the aim was to evaluate both cell behavior under dynamic and static conditions within the biomaterial and their potential for osteo-differentiation *in vitro* and *in vivo*. To the best of our knowledge, the application of human dental stem cells in 3D biomimetic scaffolds with the purpose of bone tissue engineering has not been totally explored yet.

## Materials and Methods

### Establishment of Stem Cell Cultures From the Human Dental Pulp and Follicle (hDPMSC and hDFMSC)

Human dental tissues fragments (pulp and follicle tissue) were collected, digested and MSCs were isolated by adherent culture on plastic tissue culture substrates (Supplementary Material). After confluence, cells were detached and characterized by flow cytometry and RT-PCR analysis (Supplementary Data). Human follicle and pulp MSCs were cultured in α-MEM (alpha modification of Eagle minimum essential medium, Sigma-Aldrich) with 10% fetal bovine serum (FBS, Gibco), 1% penicillin-streptomycin (3 × 10^–4^ mol/L and 5 × 10^–4^ mol/L, Gibco) and maintained at 37°C in a 5% carbon dioxide (CO_2_) atmosphere.

### Preparation of 3D Porous Scaffolds of Collagen, Nanohydroxyapatite and Phosphoserine (Coll-nanoHA/OPS) by a Cryogelation Method

Cryogels were produced as previously described (Rodrigues et al., 2013; Salgado et al., 2016, 2019). Briefly, bovine collagen Type I (Sigma-Aldrich, Germany) was homogenized (Ultra Turrax T25, IKA) at 10000 rpm, in 5 mM HCl (36.5–38% grade, Sigma-Aldrich, Germany) at a concentration of 2% (w/v). Collagen-nanoHA biocomposites were prepared by mixing the collagen solution with 1% nanoHA (particle size 5.0 ± 1.0, nanoXIM.HAp202, FLUIDINOVA, S.A, Portugal), final composition Collagen-nanoHA 50:50 w/w%). O-phospho-L-serine (OPS, ≥98% grade, Sigma-Aldrich, Germany) was added to the nanoHA suspension (0.5% w/w%) with the final mass proportion of 1:1:0.5 for the Coll-nanoHA/OPS scaffold. For the preparation of cryogels, materials were crosslinked with 10 mM of N-hydroxysuccinimide (NHS, 98% grade, Sigma-Aldrich, Germany) and 20 mM of 1-Ethyl-3-(3-dimethylaminopropyl)carbodiimide (EDC, ≥98% grade, Sigma-Aldrich, Germany), at the final mass proportion of 1:0.012:0.031 (collagen/NHS/EDC), and were kept in a freezer at −18°C for 24 h to complete the crosslinking. Afterward, materials were thawed at room temperature and the scaffolds were washed with distilled water and finally dried in a freeze-dryer (Labconco, FreeZone 6) at −80°C for 24 h.

### Cell Culture Into Biomimetic 3D Scaffolds (Coll-nanoHA/OPS)

A biocompatibility study was performed by culturing of human dental follicle and pulp mesenchymal stem cells (hDFMSC and hDPMSC) within osteoconductive scaffolds in basic or osteoinductive medium (0.1 mM dexamethasone, 0.1 mg/mL ascorbic acid and 10 mM b-glycerophosphate) that was added after 3 days of the culture. Scaffolds of collagen with nanohydroxyapatite and phosphoserine (Coll-nanoHA/OPS) were produced as described above and cut into 48 discs for each dental MSCs (5 mm × 4 mm). A total of 3 × 10^5^ cells were seeded within each scaffold (12 samples with each cell type, hDFMSC or hDPMSC) by drop method. The cells were centrifuged and concentrated in a small volume (3 × 10^5^ cells/20 μl) and dropped onto the scaffold surface. Afterward, the scaffolds were placed inside a non-tissue culture 24-wells plate for four hours and put in the standard incubator (37°C, 95% humidified air and 5% v/v CO_2_) to allow cell adhesion. Afterward, the wells were full filled (1.5 mL) with basic or osteoinductive cell culture medium and incubated for 1, 7, 14 and 21 days. These experiments aimed at evaluating hDFMSC and hDPMSC cells adhesion and viability within the biomaterials, measuring the cells proliferation and osteogenic differentiation potential, as well as determining cell morphology. Three independent experiments were performed to evaluate the cells viability and differentiation behavior.

### Dental MSCs-Loaded Coll-nanoHA/OPS 3D Scaffolds Under Static and Dynamic Conditions in the Multicompartment Holder for Spinner Flasks

Twenty-four scaffolds disks (Coll-nanoHA/OPS – 5 × 4 mm discs) loaded with hDFMSC and hDPMSC cells (3 × 10^5^ cells/scaffold) were cultured into 24 well-plate for 24 h. Later, twelve samples were transferred to a six-well culture plates (static culture - control) and the others to the multicompartment holder in a 25 mL spinner flask (dynamic conditions). Each multi-compartment holder had two containers with six independent compartments (6 mm diameter × 6 mm height) to house disk-shaped samples with a maximum size of 5 mm diameter × 5 mm height. Each individual compartment had 6 perforations of 1 mm on the top, bottom, and side, allowing fluid perfusion through 3D cell-material constructs. The container had a perforated lid to avoid free-floating samples. Under dynamic conditions, the spinner flask was kept under continuous agitation (50 rpm) with a total volume of 24 mL and, once a week the basic medium was half-renewed. The entire apparatus, including a magnetic stirring plate, was placed in a standard incubator (37°C, 95% humidified air and 5% v/v CO_2_). Static cultures in six-well plates were performed, with one scaffold per well in 4 mL of basic medium, to maintain the same volume-to-disk ratio used under dynamic conditions. Medium feeding regimen was also identical to the one used for dynamic conditions. In both cases, cell-loaded materials were maintained during 7, 14 and 21 days. At the determined the time-points, the samples were collected and cell proliferation (DNA quantification by PicoGreen assay, osteogenic differentiation by ALP activity and qPCR gene expression protocols described at section “Bone Differentiation of Human Dental MSCs in 3D Culture: Proliferation, Morphology and Differentiation.”). Cell viability was checked after 7, 14 and 21 days (LIVE/DEAD assay). Three independent experiments were performed to evaluate the scaffold biocompatibility and differentiation behavior of the cells.

### Bone Differentiation of Human Dental MSCs in 3D Culture: Proliferation, Morphology and Differentiation

#### DNA Extraction Assay

DNA content was measured using the Quant-iT^TM^ Picogreen^®^ DNA assay (Invitrogen, United Kingdom) according to the manufacturer’s instructions. Briefly, after each time point, three scaffolds were washed with PBS, they were placed at 37°C and 5% CO_2_ for 1 h with 0.5 ml of ultra-pure water. Subsequently, they were placed in a freezer at −80°C for 1 h and then thawed at room temperature to lyse all the cells membranes cultured on the materials. Finally, the fluorescence intensity was measured with a microplate spectrofluorometer (SynergyMx, BioTek) at 530 and 590 nm excitation and emission, respectively. The results are expressed in nanograms of DNA per milliliter.

#### Confocal Laser Scanning Microscope

Two samples from each time-point were fixed (4% paraformaldehyde – Sigma) and incubated for 5 min with 0.1% Triton X100 solution (Sigma), washed with 1% bovine serum albumin solution in PBS (BSA, Sigma) and the actin (cytoplasm) were stained with Alexa Fluor-conjugated Phalloidin 594 nm (Invitrogen) at 2.5% for 1 h under darkness. Nuclei were stained with DAPI (4′-6-diamidine-2-phenylindole at 0.2%, Invitrogen). Finally, the scaffolds images were acquired with a Leica SP2 AOBS SE camera, with the excitation laser of 358 and 594 nm.

#### Alkaline Phosphatase Activity and Protein Content

The same supernatant with the lysed cells obtained as described above (2.4.1) was used for the enzyme activity and total protein content protocol. ALP activity was assessed by the p-nitrophenol phosphate substrate hydrolysis (Sigma-Aldrich, Germany). After 1 h incubation at 37°C, the reaction was quantified by absorbance measurements at 405 nm, using a plate reader (Synergy^TM^ HTX, BioTek). The ALP activity results were normalized to total protein content and were expressed in nanomoles of p-nitrophenol produced per minute per mg of protein. Total protein content was measured by Lowry’s method with bovine serum albumin (Sigma-Aldrich, Germany) used as standard.

#### Osteogenic Phenotype Analyses Through mRNA Expression of Runx-2, Osteopontin (OPN), BMP-2 and Osteocalcin (OC)

Total RNA was extracted from 3 dental MSCs-loaded scaffolds at each time point with NucleoSpin kit (NucleoSpin RNA, Macherey-Nagel, Germany), as recommended by the manufacturer. Subsequently, cDNA synthesis was obtained with the iScript^TM^ cDNA Synthesis Kit (BioRad, United States) as recommended by the manufacturer. After cDNA synthesis reaction, quantitative real-Time PCR was carried out in mixture containing 1 μL of cDNA, 10 μM of each forward and reverse primers (Supplementary Table 2) and 10 μL of iTaq^TM^ Universal SYBR^®^ Green Supermix (BioRad, United States). qPCR experiments were run using an iQ5 (BioRad, United States) and analyzed with the iCycler IQ software (BioRad, United States). The housekeeping gene glyceraldehyde 3-phosphate dehydrogenase (GAPDH) was used as the endogenous assay control. Relative quantification of gene amplification by qPCR was performed using the cycle threshold (Ct) values and relative expression levels were calculated using the 2^∧^(−ΔΔCT) method. For each PCR, samples were analyzed in duplicate and three independent experiments were performed.

### Animal Model of Ectopic Intramembranous Ossification (IMO)

Twelve Coll-nanoHA/OPS scaffolds (5 × 4 mm discs) were seeded with hDFMSC or hDPMSC cells (3 × 10^5^ cells/scaffold) and cultured in a 24 well-plate for 24 h [similar as described above – section “Cell Culture Into Biomimetic 3D Scaffolds (Coll-nanoHA/OPS)”]. Afterward, samples were transferred to the multicompartment holder and cultured in the osteoinductive medium (described above) under dynamic conditions (50 rpm) for 7 days. Afterward, one control scaffold without cells, and one scaffold seeded with cells (hDFMSC or hDPMSC per scaffold) were subcutaneously transplanted into the dorso of each nude female mouse (4 animals), 6 week-old (i3S animal house, Portugal). The study was performed and approved by the Animal based studies Ethical Committee and fulfilled all legal requirements (i3S Animal Ethical Committee and DGAV, Portugal). Animals were anesthetized with 3–5% isoflurane for induction and 1–2% for surgical procedures that were performed under standard aseptic conditions. A midline incision through the dorsal skin was performed and three subcutaneous pockets were created, one on the right side (control material – without cells) and two on the left side (scaffolds with cells). The dorsal wound was then closed with surgical staples. After recovery, the mice were caged in pairs and allowed to move in their cages without restriction. They were fed with commercial mice chow and water for 8 weeks’ ad lib. After the foreseen period of time, the mice were euthanized with carbon dioxide asphyxiation. A pilot *in vivo* test was performed with two animals to evaluate the *in vitro* cell culture with osteoinductive medium and different implantation time-points (4–8 weeks) to set the final experimentation conditions.

#### Histology Analysis

All samples were explanted and fixed in 10% neutralized buffered formalin for three days and then processed for histology. Fixed samples were embedded in paraffin and were sectioned longitudinally with a microtome (5 μm of thickness). The sections were stained with Masson Trichrome and Alizarin red (calcium deposition) staining for light microscopy examination. Image analysis by the ImageJ software (Wayne Rasband) was used to determine the percentage of total tissue ingrowth area (Masson Trichrome). For the evaluation of such parameter, over 20 images were used.

#### Immunohistochemical Analysis

Immunohistochemical analysis were performed to stain the human ECM and cells. The human osteopontin (OPN) and HuNu nucleus were probed after antigen recovery. With this purpose, masked epitopes were exposed by treatment with citrate buffer (pH 9, Sigma-Aldrich, Germany) for 20 min at 97°C. Sections were incubated with mouse anti-human nuclei primary antibody (MAB4383-3 E1.3 Millipore, 1:400, United States) and rabbit anti-human osteopontin (AB 1870, Merck, 1:500, Germany). This procedure was followed by 1 h incubation with Alexa Fluor 594 goat anti-mouse IgG secondary antibody (Invitrogen Molecular Probes, 1:1000, United States) and Alexa Fluor 488 goat anti-rabbit IgG secondary antibody (Invitrogen Molecular Probes, 1:1000, United States). All slides were mounted in Vectashield^TM^ with DAPI (Vector laboratories, United Kingdom). Images were obtained using a fluorescence inverted microscope (Axio Imager Z1, Zeiss, Germany). Image analysis by the ImageJ software (Wayne Rasband) was used to determine the percentage of total human osteopontin presence (Green area). For the evaluation of over 20 images were used.

### Statistical Analysis

Data were presented as mean ± standard deviation (*n=3*) and they were analyzed using the two way ANOVA test. Differences between groups were considered statistically significant for *p* < 0.05.

## Results

### Dental MSCs Characterization and Osteogenic Differentiation

Dental follicle and dental pulp stem cells markers expressions were investigated by flow cytometry analysis. Briefly, 10^6^ cells per sample were immune-labeled to evaluate positive expressions for CD44, CD90, CD73 and lack of expressions of CD34 and CD45, that indicate a mesenchymal stem cell phenotype. Results may be seen in Supplementary Figure 1. Both cells isolated from dental follicle and pulp highly expressed the positive marker CD90. Both cell types did not express the negative marker CD34 and CD45. A very important factor in elucidating the cellular basis of tissue regeneration is determining the multipotential capabilities of stem cells to differentiate into desired target tissue. Odontogenic tissues deriving from neural crest such as hDFMSCs, showed typical features of multipotency and were characterized by a high degree of plasticity. These stemness gene expression factors were evaluated by RT-PRC (Supplementary Figure 2). Both dental MSCs showed positive expression for SCF, Thy-1, (CD90), CXCR4, and negative expression for TERT. After osteogenic induction for 21 days in the supplemented culture medium, both hDPMSCs and hDFMSC lost the mesenchymal stem cell gene expression.

In line with other published research data, this work evaluated dental stem cells capacity to induce mineralization *in vitro* and that new bone formation *in vivo* might be possible using these stem cells. Based on existing information, optimal cell culture conditions were studied and it was observed that the biocompatibility of several substrates with dental pulp or follicle stem cells induced the differentiation into osteoblasts. Exposure to osteogenic differentiation environment, such as soluble factors (ascorbic acid, β-glycerol phosphate and dexamethasone) induced osteogenic differentiation of hDPMSC and hDFMSC in this study experiments. Simple osteoinductive medium without growth factors highly increased the gene expression of osteoblast main non-collagenous proteins (osteocalcin) as shown by qPCR. The ALP activity was increased in the osteogenic differentiation medium. Although ALP is a membrane marker of all types of stem cells, it is also a marker of osteogenic differentiation. In early stage differentiation (14 days) an initial peak of ALP was observed, followed by a gradual decrease (Supplementary Figure 3).

At that stage, collagen type I was deposited in *de novo* synthesized extracellular matrix. The final stage of osteogenic differentiation (21 days) was characterized by high levels of osteogenic gene expression (osteocalcin and BPM-2), and deposition of calcium phosphate (Alizarin red and Von Kossa staining) (Supplementary Figures 4, 5).

### Dental MSCs Proliferation, Morphology and Bone Differentiation Within Biomimetic (Coll-nanoHA/OPS) 3D Scaffolds

**FIGURE 1:**
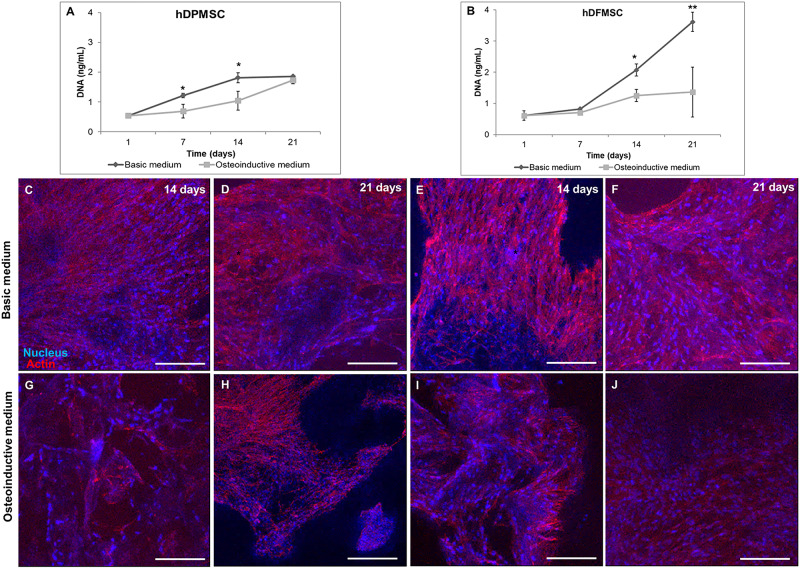
DNA concentration of hDPMSC **(A)** and hDFMSC **(B)** cultured within Coll-nanoHA/OPS for 7, 14 and 21 days in the basic vs. osteoinductive medium. Statistical analysis *p < 0.05 and **p < 0.01. CLSM maximum *Z* projection images (200 μm depth) of hDPMSC **(C,D,G,H)** and hDFMSC **(E,F,I,J)** cultured for 14 and 21 days within collagen-nanoHA/OPS biocomposite scaffolds in the basic or in the osteoinductive medium osteoinductive medium. Scale: 200 μm.

Due to their high proliferation rates and capacity to differentiate into osteoblasts, human dental stem cells offer great potential for clinical dentistry (Salasznyk et al., 2007). Recently, research on bioactive materials has been focusing in developing biomaterials with enhanced pro-regenerative potential. In this work, hDFMSC and hDPMSC were seeded within Collagen-nanoHA/OPS biocomposite scaffolds and cell proliferation was estimated by DNA quantification, as shown in Figure 1. The total DNA content of hDPMSC in 3D scaffolds for 21 days of culture in basic medium was similar to the one found when using the osteoinductive medium (Figure 1A). The same effect was not observed for hDFMSC (Figure 1B). It was possible to observe that hDFMSC exhibited higher proliferation rates than hDPMSC. Moreover, total DNA after 21 days were four fold higher in the basic medium when compared to the osteoinductive medium.

Cell distribution within Collagen-nanoHA/OPS biocomposite scaffolds (day 14 and 21) was observed by CLSM. Images (Figures 1C–J) show that dental follicle and pulp cells were well spread over all samples, entirely covering the scaffolds surfaces. CLSM images also corroborate that the scaffolds with hDPMSC have fewer cells, when compared to scaffolds with hDFMSC. Furthermore, the distribution of dental MSCs seemed to follow the irregularities of the materials’ surfaces, with cells covering the pore walls (Figures 1C–J).

**FIGURE 2:**
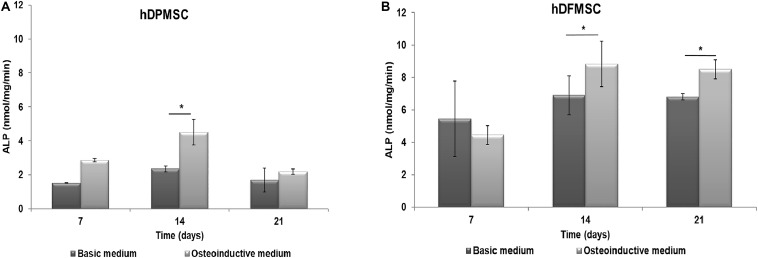
ALP activity of **(A)** hDPMSC and **(B)** hDFMSC cells cultured osteoinductive medium within Coll-nanoHA/OPS scaffolds for 7, 14 and 21 days in the basic or osteoinductive medium. Statistical analysis *p < 0.05.

The functional activity of hDPMSC and hDFMSC on Collagen-nanoHA/OPS biocomposite scaffolds was assessed by measuring the ALP activity after culture for up to 21 days. ALP produced by the cells was normalized to the total protein content, and results were expressed in nmol/min/ng, as shown in Figure 2. hDPMSC cultured on the biomimetic scaffold showed higher ALP activity under osteoinductive than under basic conditions, for all time points, but the difference was only statistically significant at day 14 (Figure 2A). However, hDFMSC seeded on Collagen-nanoHA/OPS biocomposite scaffolds exhibited significantly higher ALP levels after 14 and 21 days of culture in osteoinductive medium, and the double of activity when compared to hDPMSCs (Figure 2).

**FIGURE 3:**
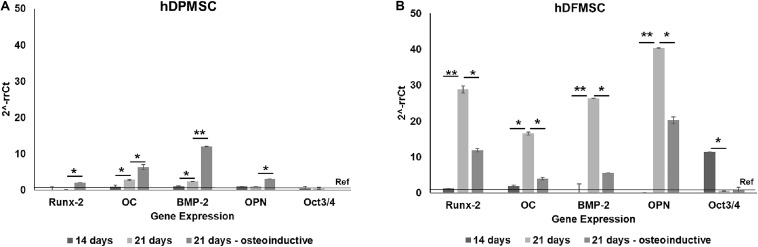
Analysis of the expression of osteogenic genes (Runx-2, osteocalcin, BMP-2 and osteopontin) and mesenchymal gene (Oct3/4). Quantitation of data was performed using the ΔΔCt method using GAPDH gene expression as an endogenous reference. Results were normalized to the undifferentiated dental cells (passage 6) average results, and are represented as fold change. **(A)** hDPMSC cells and **(B)** hDFMSC cells cultured for 14 and 21 days (in the basic or in the osteoinductive medium osteogenic induction) within Coll-nanoHA/OPS scaffolds. Statistical analysis *p < 0.05 and **p < 0.01.

In accordance to ALP activity results, the quantitative PCR evaluation after 21 days showed expression of several osteoblast-associated markers by dental MSCs in both osteoinductive and basic culture medium (Figure 3). The hDPMSC seeded on scaffolds under basic conditions showed a 2-fold enhancement for Runt related transcription factor 2 (Runx-2) and bone morphogenetic protein type 2 (BMP-2); and 6-fold for osteocalcin (Figure 3A). The hDFMSC showed a 30-fold change for Runx-2 and BMP-2 expressions, and 40-fold for OPN (Figure 3B). Both dental MSCs lost the expression of the multipotency stem cell gene Oct3/4 (transcription factor for MSC) after osteogenic differentiation (Figures 3A,B).

### Cell-Loaded 3D Scaffolds Under Static and Dynamic Conditions in the Multicompartment Holder for Spinner Flasks

**FIGURE 4:**
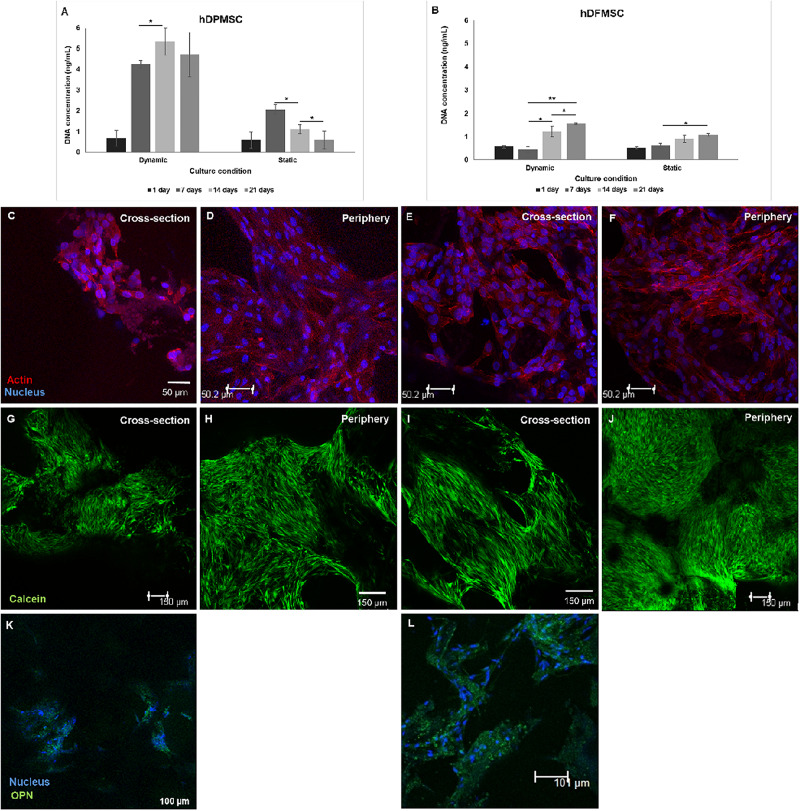
Proliferation (DNA concentration) of hDPMSC **(A)** and hDFMSC **(B)** cultured within Coll-nanoHA/OPS for different time points (7–21 days) in static vs. dynamic conditions. Statistical analysis *p < 0.05 and **p < 0.01. CLSM images of dental MSCs cells (Pulp – **C–D**, Follicle – **E–F**) cultured for 21 days. Cross-section of the material **(C,E,G,I)** and of the periphery **(D,F,H,J)** within collagen-nanoHA/OPS scaffolds under dynamic culture. Scale: **(C–F)** 50 μm and **(G–J)** 150 μm. Human OPN immunostaining (Green: **(K,L)**, scale: 100 μm). Blue – Nucleus, Red – cytoskeleton (actin), Green – calcein and red - propidium iodide.

Dynamic conditions were established using spinner flasks under 50 rpm agitation speed, equipped with an in-house designed multicompartment holder that protects the scaffolds from damage (Teixeira et al., 2014). Both types of dental MSCs cultured within Coll-nanoHA/OPS 3D scaffolds were able to survive, actively proliferate, and migrate throughout the scaffolds depth, under dynamic conditions. Under dynamic conditions, after 14 days hDPMSC showed higher DNA concentration and an increase in cell numbers, that remained similar after 21 days. Different proliferation rates were observed under static conditions, the hDPMSC number decreased over time (Figure 4A). Dental follicle MSCs proliferation was confirmed by the increase in DNA content after 14 days, although the same proliferation rate was not observed under static conditions, even after 21 days (Figure 4B). In both cell culture conditions, DNA concentrations in hDFMSC cultures were 4 times lower than in hDPMSC cultures. Yet, after 21 days under static conditions, hDFMSC showed higher DNA concentration then hDPMSC.

By the CLSM imaging, dental MSCs were observed at the scaffolds’ periphery and cross-section, both under dynamic and static conditions. hDPMSC cross-section images showed lower cell numbers under dynamic conditions, when compared to the scaffolds’ periphery area (Figures 4C,D). In both culture regimens (static and dynamic), cells remained viable on the materials’ periphery and throughout the cross-section, but hDFMSC showed better and more homogeneous spatial distribution of viable cells (calcein positive) in the 3D scaffold (surface and center) after 21 days of culture (Figures 4E,F). These observations are in agreement with the live/dead assay images that showed high numbers of viable cells with a homogeneous distribution within the 3D structures (Figures 4G–J). After 21 days, both types of dental MSCs showed OPN secretion, suggesting osteogenic differentiation, under dynamic culture in basic medium (Figures 4K,L). At the same time point, hDPMSC showed lower OPN deposition, when compared to hDFMSC, but both cell types showed protein accumulation at the materials periphery.

**FIGURE 5:**
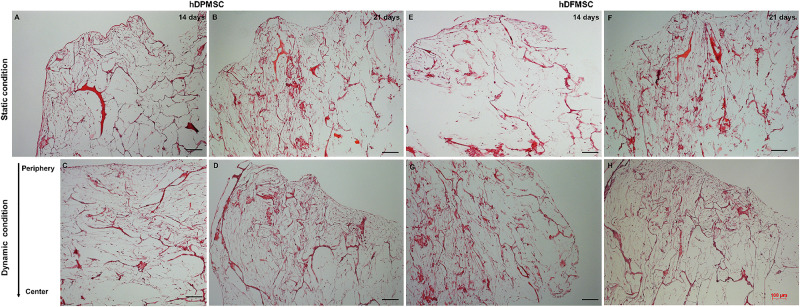
Optical microscopy images of dental MSCs (hDPMSC: **A–D** and hDFMSC: **E–H**) within Collagen-nanoHA/OPS scaffolds under static vs. dynamic conditions for 14 and 21 days. Slides were stained using H&E. Scale: 100 μm.

Histology sections of these samples showed differences in the cell presence at the surface and at the inner region of the scaffold. Both MSC types cultured under static conditions showed higher presence of cells at the surface and an empty scaffold’s inner region after 14 and 21 days (Figures 5A,B,E,F). On the contrary, hDPMSC and hDFMSC cultured under dynamic conditions showed better cell distribution within the scaffolds for both time points (Figures 5C,D,G,H).

**FIGURE 6:**
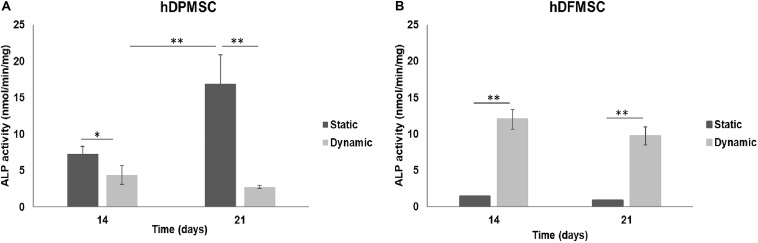
ALP activity on human dental follicle and pulp MSCs cultured within Coll-nanoHA/OPS for different time points (14 and 21 days) under static vs. dynamic conditions. Statistical analysis ^∗^*p* < 0.05 and ^∗∗^*p* < 0.01.

The potential of both dental MSCs to undergo osteogenic differentiation was further explored by analyzing ALP activity and osteogenic gene expressions. In Figure 6, at comparing the two tooth-delivered cells, hDPMSC showed three-fold higher ALP activity under static conditions after 21 days. Importantly enough, hDFMSC, showed a 2-fold higher ALP activity in dynamic 3D cultures after 14 days, but pulp cells showed reduction in the ALP activity after 21 days. Still, follicle MSCs had the lowest enzyme activity under the same conditions (Figure 6).

**FIGURE 7:**
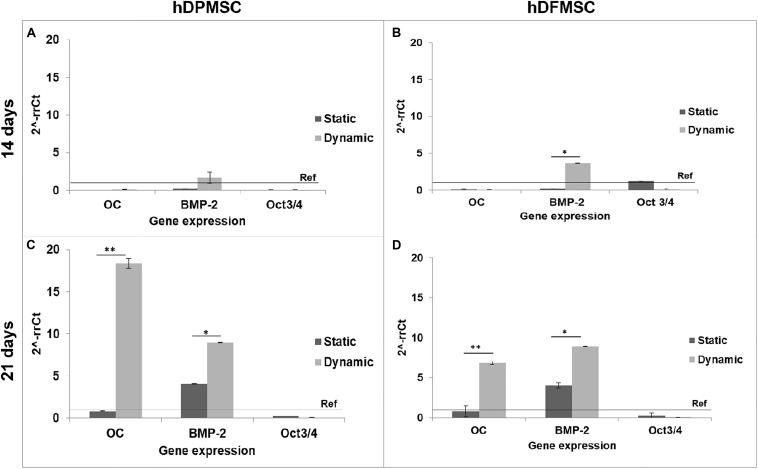
Quantitative real-time polymerase chain reaction (qPCR) for osteogenic genes (Osteocalcin and BMP-2) and mesenchymal gene (Oct3/4) for dental pulp and follicle MSCs cultured within the scaffold for 14 and 21 days under static vs. dynamic conditions. Quantitative data were calculated by the ΔΔCt method using GAPDH gene expression as an endogenous reference. Samples results were normalized to the undifferentiated cells (passage 6) average results. These are represented as fold change. Statistical analysis ^∗^*p* < 0.05 and ^∗∗^*p* < 0.01.

The results of gene expression related to osteogenic differentiation, showed that hDFMSC presented an early peak of BMP-2 expression after 14 days under dynamic conditions, when compared to the static control. After 21 days, there was an 18-fold increase on osteocalcin expression for hDPMSC, and a 7-fold for hDFMSC (Figure 7). A similar enhancement in the BMP-2 expression was also observed for both dental MSCs (8-fold change).

### Animal Model of Ectopic Intramembranous Ossification (IMO)

**FIGURE 8:**
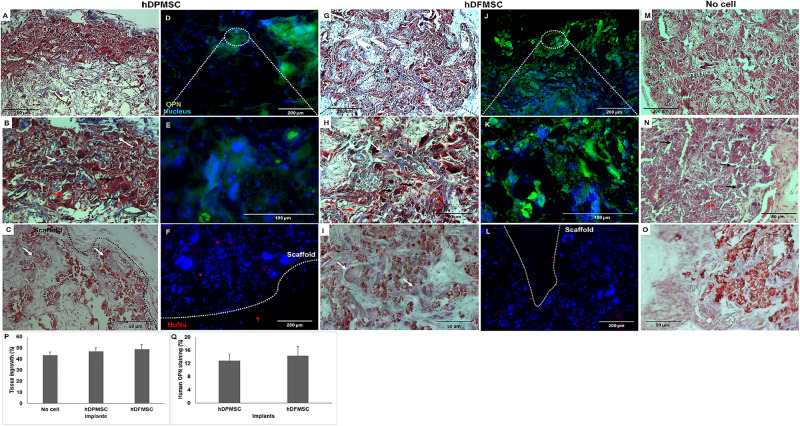
Optical microscopy images of dental MSCs (hDPMSC – **A**,**B**,**C** and hDFMSC – **G**,**H**,**I**) within Collagen-nanoHA/OPS scaffolds implanted for 8 weeks. Slides were stained by Masson Trichrome **(A,B,G,H)** and Alizarin red **(C,I)**. **(M,N)** Implants of scaffolds without cells after 8 weeks. **(O)** Implants without cells were stained with Alizarin red. Black dashed line - scaffold surface, white arrows – bone-like tissue, red arrows - new blood vessels and black arrows - giant cells. Scale: **(A,G,M)** 200 μm and **(B,C,H,I,N,O)** 50 μm. Fluorescence microscopy images of dental MSCs (hDPMSC – **D**,**E**,**F**,hDFMSC – **J**,**K**,**L**) within collagen-nanoHA/OPS scaffolds and implanted for 8 weeks. Blue – Nucleus (DAPI), Green – Human Osteopontin, Red – human nucleus (HuNu), White dashed line – scaffold surface. Scale: **(A,F,J,L)** 200 μm and **(E,F)** 100 μm. **(P)** 3D Coll/nanoHA scaffolds with and without human dental MSCs (percentage of the total section area). **(Q)** Human OPN presence within the scaffolds with hDPMSC or hDFMSC.

3D scaffolds seeded with both dental cell types were cultured under dynamic conditions and osteogenic medium for 7 days, and then implanted subcutaneously in immunocompromised mice. These scaffolds under osteoinduction medium were characterized by DNA concentration, ALP activity and osteogenic gene expression to compare with the results for basic medium, although the results showed no statistically significant difference between the culture mediums (Supplementary Figure 6). In a previous work (Salgado et al., 2019) and according to the literature (Scotti et al., 2013; Stüdle et al., 2018), the *in vivo* experiment was performed with 3D cultures in the presence of the osteoinductive medium. Dynamic culture conditions were chosen, as they enhanced the dental follicle MSCs migration into the inner part of the scaffold, favoring mass transfer and oxygenation, avoiding the hypoxia-necrosis effect of *in vitro* engineered tissues. Both cellularized 3D constructs (with hDPMSC and hDFMSC) showed significant tissue ingrowth after 8 weeks (Figure 8). Higher number of multinucleated cells (giant-cells) could be observed next to the 3D scaffolds surface (Figures 8A,G). The results also showed that the materials seeded with MSCs induced the continuous growth of the surrounding tissue inside the porous scaffold. In the control samples (scaffolds without cells – Figures 8M–O) of the *in vivo* subcutaneous model, it was possible to observe the presence of giant cells and inflammatory cells such as macrophages surrounding the apatite particles (Figure 8N). Nanohydroxyapatite particles could be observed by Alizarin red staining (red dots – Figure 8O), but calcium deposits could not be observed within the scaffold. The total tissue area within the artificial 3D scaffolds were calculated with the Image J software and plotted as a total percentage. It was observed that the presence of both dental MSCs enhanced the percentage of the total tissue ingrowth after 8 weeks, but they were not statistically different from the material without the human cells (Figure 8P).

At the periphery of the implant, a bone-like structure surrounding the nanohydroxyapatite particles could be observed (Figures 8B,H). Nanohydroxyapatite particles and tissue calcium deposition could be seen with Alizarin red staining. This histochemical analysis showed that these tissue areas were positive for the mineralization staining (Calcium deposits – Figures 8C,I). According to the Masson trichrome staining, the bone-like structures (red color – calcium deposits) were more intensely present in the periphery of the scaffold, which could be directly related to the *in vitro* dental MSCs seeding cells in the presence of the osteoinductive medium for 7 days (Supplementary Figure 8).

After immunostaining human cells inside the implants, it could be observed that in all the cell-loaded scaffolds implanted for 8 weeks, small amounts of both human dental MSCs were still present inside the materials. The number of dental cells inside the biocomposites after 60 days was considerably lower and the outward migration/invasion of these cells into adjacent mouse tissue was not remarkable (Figures 8F,L). The human proteins secreted by the dental MSCs were evaluated by the detection of human OPN inside the scaffold (Figures 8D,E,J,K). hDPMSC showed OPN accumulation at the periphery of the scaffold (Figures 8D, 9E). On the contrary, the presence of OPN was more evident in hDFMSC-loaded scaffolds, and the protein was homogeneously distributed throughout the porous structure (Figures 8J,K), in accordance with the more significative presence of human cells in that region (Figure 8L). Eight weeks after implantation, newly synthesized ECM with human OPN area was quantified by the Image J Software, but, the human protein presence did not show statistical difference between hDPMSC and hDFMSC-loaded scaffold implants (Figure 8Q).

## Discussion

Access to tooth-derived stem cells is comparatively easier, if compared to other sources, produces very low patient morbidity and cell extraction is highly efficient. In addition, dental mesenchymal cells have good osteogenic differentiation ability and present high capacity to adhere to biomaterials’ surface, thus making them a promising source for bone tissue regeneration (Graziano et al., 2008). The search for alternative sources of MSC(such as the dental follicle and pulp) for bone regeneration is of considerable importance since bone marrow-derived MSCs show significant age-related decrease in frequency and differentiation potential (Brady et al., 2014). Compared to other stem cell sources from the oral cavity, tooth pulp and follicle are considerably large tissues (Mori et al., 2012), easier to access, and with higher proliferation capacity (Shoi et al., 2014).

A very important factor in elucidating the cellular basis of tissue regeneration is determining the multipotential capabilities of stem cells to differentiate into the desired target tissue (Chambers et al., 2003). Odontogenic cells deriving from neural crest, such as dental follicle stem cells, show typical features of multipotency and are characterized by a high degree of plasticity, with capacity to differentiate into cell lines derived from two germ layers (i.e., osteoblasts and adipocytes, as well as neuronal cells) (Dominici et al., 2006). The objectives of this work was to combine three experimental series to evaluate (i) the follicle and pulp dental MSCs capacity to differentiate into osteogenic lineage under supplementation of osteogenic factors in the culture medium within a biomimetic scaffold based on type 1 collagen, nanohydroxyapatite and phosphoserine; (ii) the hydrodynamic and mass transfer conditions that could influence the tooth-derived MSCs osteogenesis in a 3D scaffold; (iii) to clarify the viability, migration and osteogenesis within a 3D scaffold of the two different dental MSCs with a pre-clinical animal model that is predictive and translational such as an ectopic ossification in rodents.

In this work dental follicle and dental pulp stem cells markers expression were studied by flow cytometry analysis (Supplementary Material). Both cells isolated from dental follicle and pulp expressed the positive mesenchymal surface markers CD90, 44 and 73, as well as the absence of expression of the negative markers CD34 and CD45. RT-PCR results after 21 days *in vitro* culture confirmed the dental MSCs commitment to the osteoblastic lineage, with high expression of several osteoblast-associated gene expression markers and decrease of undifferentiated stem cell transcriptional markers (Supplementary Figure 1).

At present, there is a particular interest in the role of dental stem cells application to bone regeneration, in particular dental pulp and follicle MSCs (Huang et al., 2009; Mori et al., 2012; Zhang and Cheng, 2013; Bojic et al., 2014; Kim et al., 2019; Lee et al., 2019). In line with other published documents, this work showed that hDPMSC and hDFMSC could be induced to differentiate into osteoblasts *in vitro* (osteogenic induction). Therefore, after 21 days of osteoinduction, the dental pulp and follicle MSCs showed higher ALP activity and enhanced osteogenic gene expression (osteocalcin and BMP-2) when compared to the same cells cultured in basic medium (Supplementary Figure 3).

Composite materials reinforced with calcium phosphate ceramics showed a higher mechanical stability and a much slower degradation rate when compared to simple polymeric materials. This type of scaffolds has shown osteoconductive properties to MSCs which expressed osteoblast-like gene markers (McCafferty et al., 2014). Also, recent studies showed that nanohydroxyapatite integrated in scaffolds was capable to recruit bone marrow MSCs and promote their osteogenic differentiation (Rodrigues et al., 2013; Salgado et al., 2016, 2019). In this work, the results illustrated the ability of dental MSCs cultured in biocomposite cryogels to differentiate into osteoblastic cells, with high ALP enzyme activity and bone phenotype gene expression. The biomimetic scaffold induced the osteogenic differentiation without osteinductive medium and enhanced the differentiation capacity of the dental-derived cells between days 14 and 21 while decreasing their levels of proliferation. These results are in accordance with previous works showing that biomaterials combined with dental MSCs under osteoinductive medium improved osteogenic differentiation (Woloszyk et al., 2014; Vecchiatini et al., 2015). RT- PCR studies indicated that Runx-2 and ALP were up-regulated on both MSCs (from dental follicle and pulp tissue). Runx-2 expression is necessary for multipotent MSCs to differentiate along the osteoblastic lineage and its level of expression is increased during osteoblast differentiation, during the mineralization phase. Thus, Runx-2 overexpression upregulates ALP activity and the expression of osteoblast-specific genes such as osteocalcin (OC) and osteopontin (OPN) (Ducy et al., 1997; zur Nieden et al., 2003; Stein et al., 2004; Mikami et al., 2007; Datta et al., 2008; Shoi et al., 2014). The significantly higher level of OC expression, a late osteogenic marker, was shown by the dental pulp MSCs when compared to the basic culture medium reference. This result indicated that these cells in the 3D construct were in a later phase of osteogenic differentiation, presenting features of mature osteoblasts and matrix mineralization. However, hDFMSC showed overexpression of BMP-2 that is directly involved on the increased gene expression of Runx-2 that strongly promotes MSCs osteogenic differentiation (early pre-osteoblast differentiation) (Carreira et al., 2014).

The method to culture 3D scaffolds in spinner flasks used in this work had the advantage of providing a dynamic environment in 3D, while protecting the structures from mechanical damage, without requiring complex approaches for holding and securing the samples. Acceptable levels of shear stress are a critical parameter in dynamic culture of mammalian cells. Usually, high agitation speeds (80 rpm) of spinner flasks result in decreased cell viability when compared to low agitation speeds (45 rpm), due to shear stress cell damage resulting in cell death (Chen et al., 2013), particularly for MSCs culture in polymeric scaffolds (Kim et al., 2007; Cetin et al., 2012). The results obtained for pulp MSCs showed lower proliferation rate after 7 days (Figures 4A,B). The shear stress seemed to affect the hDPMSC proliferation rate, but the cells remained viable after 21 days (Figures 4, 5). On the contrary, hDFMSC proliferation rate confirmed the good performance of the dynamic conditions with the multicompartment holder, since an increase in DNA content was observed after 14 days, which was not observed under static conditions even after 21 days. However, under static condition (6 well-plate), the cells showed a lower proliferation rate (lower DNA concentration – Figures 4A,B), when compared to the results from static culture (24 well-plate) in Figures 1A,B. The lower basic media volume in the first experiment (1.5 mL) resulted in the media being exchanged twice a week, instead of once a week on the 6-well plate experiment. The higher basic medium refresh revealed a positive effect on the hDFMSC proliferation rate.

One outstanding feature of this dynamic system for cell-loaded materials culture is its clear effect on spatial cell distribution and higher viability inside 3D structures (Figure 5). The dynamic conditions used in this study may offer a more favorable hydrodynamic environment, providing the necessary physical stimuli and nutrient transport to support tissue development (Bilgen et al., 2005; Bilgen and Barabino, 2007). However, it was clear by the results obtained that human dental MSCs from different tissue sources showed different spatial distribution. The hDPMSC showed intense cellular presence at the periphery of the scaffold, but with the scaffold cross-section remaining almost empty (Figures 4C,D, 5A–D). Previous studies indicated that the application of mechanical loading (turbulent flow) accelerates the process of hDPMSCs differentiation and mineral deposition while reducing their proliferation activity (Woloszyk et al., 2014; Marrelli et al., 2018). But, it was observed that only the hDFMSCs under dynamic culture within the 3D scaffolds were well-distributed through the materials porous structure (Figures 4E,F, 5E,F), showing higher osteogenic differentiation capacity (higher ALP activity and enhanced expression of osteogenic markers).

The mice model of subcutaneous implants for ectopic bone formation should allow for the evaluation of transplanted human cells in terms of viability, proliferation, migration and osteogenic differentiation capacity. For this work, 8-week duration period was the time selected to assess the new bone formation ability of human cells in a scaffold, and evaluate the long-term inflammatory response. Histology analysis revealed that some of the nanohydroxyapatite particles remained in the tissue, but the bovine collagen was no longer detectable at that time point. The maintenance of the nanohydroxyapatite must be related to its low biodegradation rate, since these were sintered particles composed by aggregates of nanocrystals strongly bond to each other. Besides, the histological findings support that the cell-loaded scaffold enhanced animal tissue ingrowth and angiogenesis. Importantly enough, newly formed extra cellular matrix stained for human OPN and a bone tissue-like structure with calcium deposition was also observed (Figures 8C,I). This bone-like tissue formation was progressively decreasing as it went from the periphery toward the center of the scaffolds. This may be related to the dynamic flow from inside the spinner flask and the fact that higher numbers of dental MSCs were adhered on the materials’ surface after 7 days of *in vitro* culture (Supplementary Figure 7). Even in intra-bone critical defects, implants did not show the formation of mature and organized trabecular bone structure after the 8 weeks of implantation. Usually, in ectopic bone formation models, the bone tissue growth should be observed after 12 weeks (Scotti et al., 2013), but in a previous work, evidence of mineralized tissue was observed in a study after only 4 weeks post-implantation, suggesting that the employed scaffold had an osteoinductive effect and promoted bone tissue ingrowth (Salgado et al., 2019). To track the transplanted cells, immunostaining for human nucleus was performed, allowing to observe a few human dental MSCs at the periphery of the scaffold, which showed some migration capacity toward the animal surrounding tissue (Figures 8F,L). These cells were apparently viable and secreted human osteopontin. The presence of human OPN was only observed inside the cell-loaded implants (dental pulp or follicle MCSs) and not in the empty implanted 3D scaffolds.

## Conclusion

In this work, it was shown that isolated dental follicle and pulp stem cells meet the necessary criteria to be named as MSC and present remarkable osteogenic potential. This work proved that both dental MSCs exhibited a progressively *in vitro* proliferation, high cellular viability and osteogenic differentiation within the 3D biomimetic scaffolds during the period of observation. When combined, the biomimetic scaffold and the dental follicle MSCs promoted high cellular proliferation rate, while cells remained viable and well-distributed within the 3D biocomposite structure and osteoblast-like cell phenotype gene expression and high ALP activity were found. But, only hDFMSC behavior was more improved under dynamic conditions. *In vivo* studies showed a disorganized subcutaneous tissue ingrowth with the observation of a bone-like structure on the materials periphery, showing a desirable hDFMSC and hDPMSC differentiation into bone tissue. In addition, these dental follicle tissues proved to be an excellent source of stem cells with a controlled and reproducible differentiation behavior to be applied in bone tissue engineering. In the future, these MSCs shall be tested in pre-clinical animal models with critical bone defects to observe their potential to promote bone tissue regeneration.

## Data Availability Statement

All datasets presented in this study are included in the article/Supplementary Material.

## Ethics Statement

The animal study was reviewed and approved by Ethical Animal Commission of Instituto de Investigação e Inovação em Saúde (i3S), Universidade do Porto (UPorto), Portugal.

## Author Contributions

CS substantially contributed to the conception and design of the biocomposite, biological and animal experiments, performed all acquisition, and analysis and interpretation of data. CS agreed to be accountable for all aspects of the work in ensuring that questions related to the accuracy or integrity of any part of the work are appropriately investigated and resolved. CS prepared and revised the draft critically for important intellectual content. CB contributed to the analysis and interpretation of data and critically revised the draft for important intellectual content and gave CS final approval on the version to be published. FM revised the draft critically for important intellectual content and gave his final approval of the version to be published. All authors contributed to the article and approved the submitted version.

## Conflict of Interest

The authors declare that the research was conducted in the absence of any commercial or financial relationships that could be construed as a potential conflict of interest.
