# Supplementary material for: Clarifying the Tooth-Derived Stem Cells Behavior in a 3D Biomimetic Scaffold for Bone Tissue Engineering Applications
Source: Front Bioeng Biotechnol. 2020 Jun 26;8:724. doi: 10.3389/fbioe.2020.00724 (PMC7333018; doi:10.3389/fbioe.2020.00724)
Supplement: Supplementary file 2 [file Data_Sheet_2.pdf]

Supplementary Material

1. Supplementary Data

1.1. Harvesting the dental follicle

Dental follicle was surgically removed from the completely intra-bone impacted third molar. The follicular sac was harvested during molar odontectomy. These surgeries were performed by the same oral surgeon with many years of experience. Subjects included in our research had age comprised between 21-25 years. Patients had a total intra-bone molar inclusion. The teeth were free of infectious complications, cysts or tumors. The patients confirmed the participation in this study by signing an informed consent declaration. The clinical protocol of the tissue collection was evaluated by an Ethical Committee of University of Porto (50/CEUP/2018, Porto, Portugal). In order to compare the dental follicle stem cells characteristics, the dental pulp was also removed from the same impacted teeth.

1.2. Establishment of Stem Cell Cultures from the Dental Follicle (hDFMSC) and Dental Pulp MSCs (hDPMSC)

Fragments from dental follicles were placed in Falcon tubes containing complete medium (αMEM, 10% inactivated fetal bovine serum (FBS - qualified from U.S.A., Gibco), 1% antibiotics, 1% fungizone, all reagents were obtained from Sigma-Aldrich, Germany). Another fragment was fixed in 10% formaldehyde for histological studies. Dental follicle fragments were cut in small pieces and an enzymatic digestive cocktail was used: 0.1% collagenase I (from *Clostridium histolyticum*, ≥125 CDU/mg solid, Sigma-Aldrich, Germany) + 0.25% trypsin EDTA (Sigma-Aldrich, Germany). After the digestion process, tissue fragments were washed with complete medium and cell suspensions were filtered and centrifuged to obtain a monolayer cellular suspension. After 24 hours, the first adhered cells should appear and after 3 days, when cell monolayers were confluent, the cells were removed and frozen. Cells showed a fibroblastic shape. Similar protocol was done to select and characterize the dental pulp stem cells (hDPMSC).

Stem cells markers expression was investigated by flow cytometry analysis. Briefly, 10^6 cells per sample were immune-labeled to evaluate positive expression for CD73, CD44, CD90, and negative expression for CD34, CD45 (all from ImmunoTools, Germany).

Cells were analyzed with BD FACS Canto II, 6-color flow cytometer, using BD FACS Diva version 6.1.3 software.

1.2.1. RT-PCR analysis

Expanded cells were analyzed for gene expression: TERT, SCF, Thy-1, (CD90), CXCR4 (Supp. Table 1).

Supplementary table 1: Primers for PCR amplification.

Gene	Primer Sequence (Forward)	Primer Sequence (Reverse)
GAPDH	5'-TAACTGGTAAAGTGGATATTG-3'	5'-GAAGATGGTAGATGGATTTC-3'
TERT	5'- CATCATCAAACCCCAGAACAC-3'	5'- CAAACAGCTTGTTCTCCATGT-3'
SCF	5'-TATTAATCCTCTCGTCAAAAC-3'	5'-AGAATTCTTCAGGAGTAAAGAG-3'
Thy-1	5’-AAAGAAGCACGTGCTCTTTGGC-3’	5’-ACTCAGAGAAGTAGGATCTCTG3’
CXCR4	5’-ATTCCTTTGCCTCTTTTGCAGATATA-3’	5’-ATGGCCAGGTAGCGGTCCAGACTGATGAA-3’

Briefly, total RNA was extracted from cell culture on material using the NucleoSpin RNA (Macherey-Nagel, Germany) kit according to the manufacturer’s instructions and subjected to reverse transcriptase polymerase chain reaction (RT-PCR) amplification (Titan One Tube RT-PCR system; Roche, Branchburg, NJ) for 30 cycles. RT reaction mixtures consisted of extracted RNA, Titan RT-PCR buffer, dithiothreitol, deoxynucleoside triphosphate, primers for each gene tested, reverse transcriptase enzyme (PCR) and water in a total volume of 25 µL. Total RNA was reverse transcribed with PCR-RT, at 60 °C for 30 min, followed by a 2-min denaturation at 94 °C. The complementary DNAs (cDNAs) were then amplified with recombinant Taq-DNA polymerase under the following conditions: 30 cycles of denaturation (94 °C/30 s), annealing (55-60 °C/30 s), elongation (68 °C/45 s), followed by a prolonged elongation of 7 min at 68 °C. Supplementary table 2 shows the primer sequences used for PCR amplification. To obtain a semi-quantitative assessment of gene expression, data were expressed as normalized ratios by comparing the integrated density values for all genes tested with those for glyceraldehyde-3-phosphate dehydrogenase (GAPDH). The PCR products were separated by 1% agarose gel electrophoresis and visualized by GelRed™ Nucleic Acid Stain (Biotium). The images of the gel were captured with a camera and analyzed with Image J software.

Supplementary table 2: Primers for PCR amplification.

Gene	Primer Sequence (Forward)	Primer Sequence (Reverse)
GAPDH	5'-TAACTGGTAAAGTGGATATTG-3'	5'-GAAGATGGTAGATGGATTTC-3'
Runx-2	5'-GTGCCTAGGCGCATTTCA-3'	5'-GCTCTTCTTACTGAGATGGAAGG-3'
OC	5'-AGAGTCCAGCAAAGGTGCAG-3'	5'-TCAGCCAACTCGTCACAGTC-3'
BMP-2	5'-GACGAGGTCCTGAGCGAGTT-3'	5'-GCAATGGCCTTATCTGTGAC-3'
OPN	5'-ACTCGAACGACTCTGATGATGT-3'	5'-GTCAGGTCTGCGAAACTTCTTA-3'
Oct 3/4	5'-AGGAGTCCCAGGACATCAAAG-3'	5'-TCGTTTGGCTGAATACCTTC-3'

1.2.2. Inducing differentiation into osteogenic lineages and immunocytochemical staining.

A differentiation protocol using basal and complex osteogenic differentiation medium were performed until the 6th passage. Cells were seeded in culture 24-well plate for induction of osteogenic differentiation in vitro. For the osteogenic medium, the basic medium was supplemented with 10 mM β-glycerophosphate, 0.0l mg/mL L-ascorbic acid, and 10^{-8} M dexamethasone.

1.2.3. Histocytochemical staining

Histocytochemical staining was performed after 3 weeks of culture as described above in section 1.2.2. Briefly, for ALP staining, fixed cultures (4% paraformaldehyde, Sigma-Aldrich, 10 minutes) were incubated with sodium naphthyl phosphate and Fast Blue RR Sigma-Aldrich, St Louis, MO (pH = 10, 1 hour), ALP stained brown to black color. Phosphate deposits were assessed by the von Kossa assay. The cultures were covered with a 1.0% silver nitrate solution and kept for 1 h under an UV light. After the samples had been washed with distilled water, a 5.0% sodium thiosulfate solution was added for 2 min and cultures were washed again. Phosphate deposits stained black. Alizarin red staining was used to assess calcium-rich deposits produced by cells in culture. A pH 6.3 solution was produced by mixing 3 mg of Alizarin Red S with 3 mL of 0.28% NH_4OH. This solution was filtered and applied to the fixed cells for 2 min. Afterward, cells were washed with distilled water, and a solution of acidic ethanol was added for 15 s. Cells were again washed and left to dry. The reaction stained the calcium deposits orange/red. For Collagen Type I staining, 1% picric acid solution and picosirius red solution were used. Cells were covered by picosirius red solution for 1 hour. Then cells were washed

with HCl solution (10mM). Collagen type I were stained in red (all reagents were provided by Sigma-Aldrich, Germany). After drying the staining, the cells were evaluated by Inverted Optical microscopy (Light microscope Olympus DP 25).

2. Results

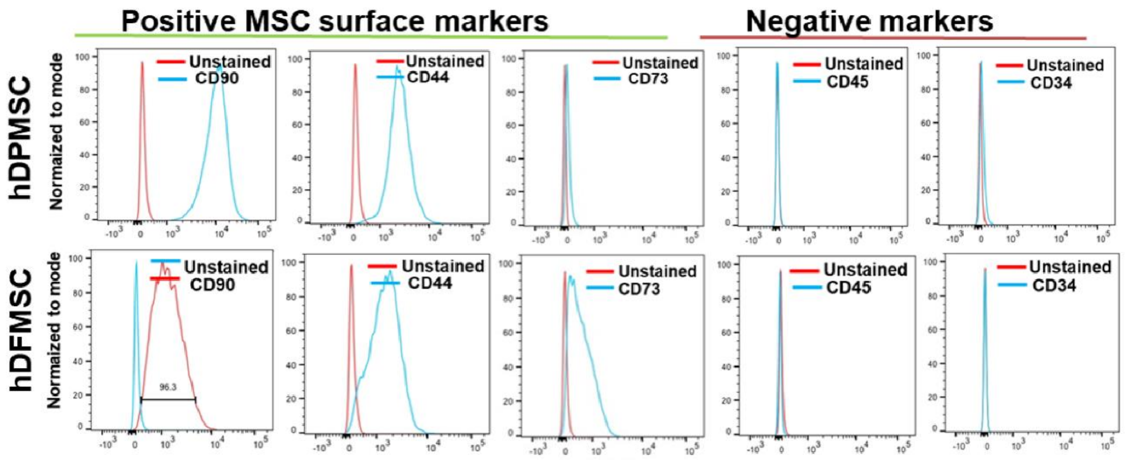

Supplementary Figure 1: Characterization of surface marker profiles for hDPMSC and hDFMSC. The flow cytometry results revealed that the hDPMSC and hDFMSC were positive for CD90, CD44, and CD73, but negative for CD34 and CD45.

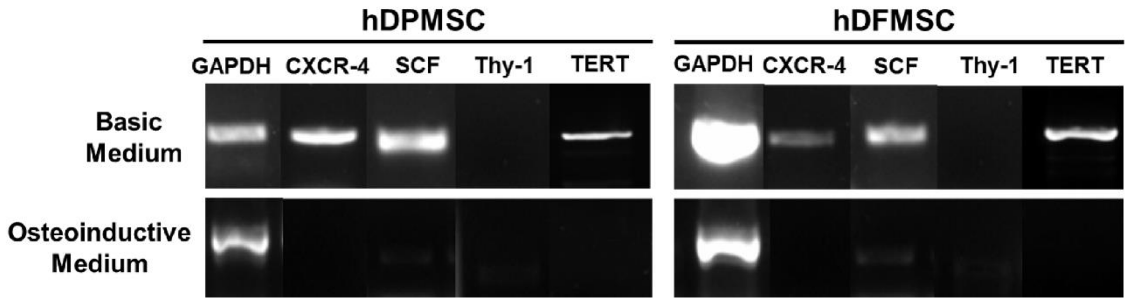

Supplementary Figure 2: Human dental pulp mesenchymal stem cells (hDPMSC) and human dental follicle mesenchymal stem cells (hDFMSC) mesenchymal stem gene expression markers before and after the osteogenic induction.

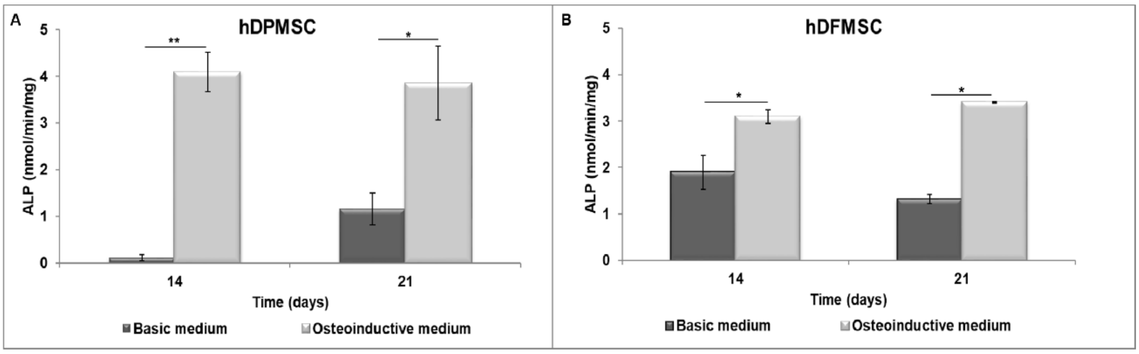

Supplementary Figure 3: ALP activity of (A) dental pulp mesenchymal stem cells (hDPMSC) and (B) dental follicle mesenchymal stem cells (hDFMSC) cells cultured with or without osteogenic induction for 14 and 21 days.

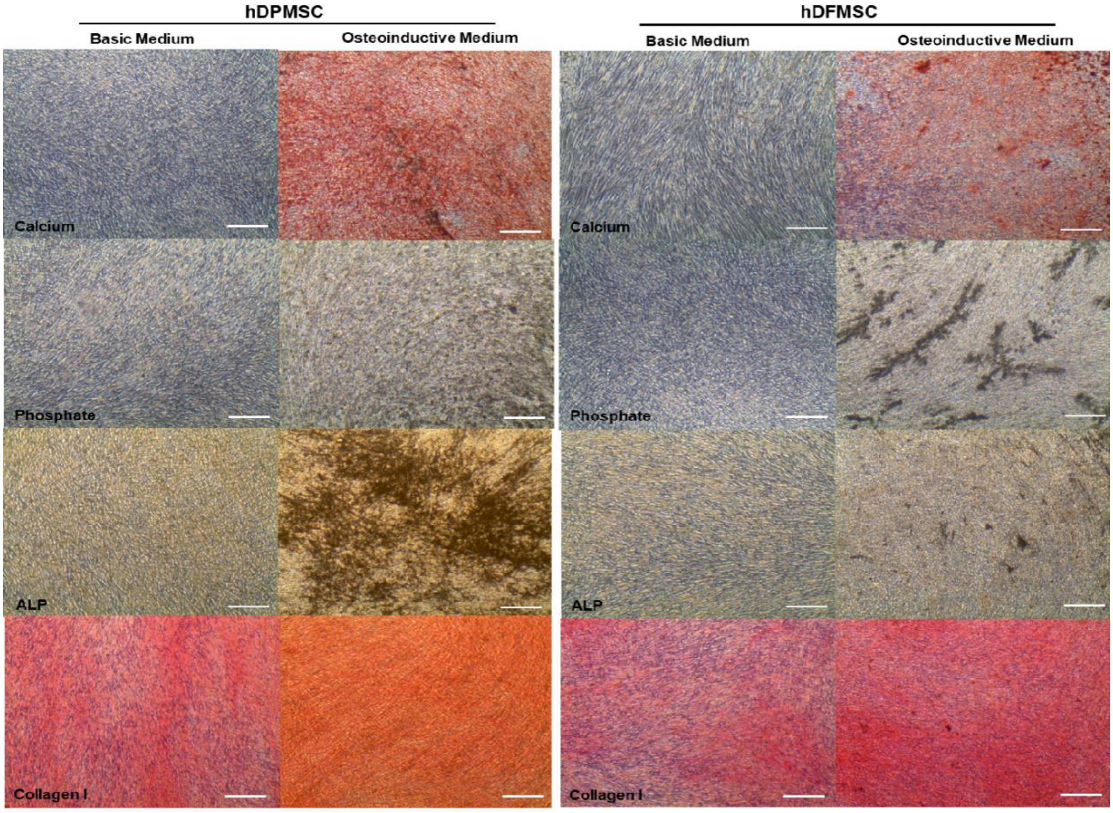

Supplementary Figure 4: Histomorphometric analysis for osteogenic differentiation of the human pulp cells (hDPMSC) and follicle cells (hDFMSC) cultured in osteogenic medium for 21 days. Alizarin red – calcium deposits, ALP – alkaline phosphatase activity, Von Kossa – Phosphate deposits and Sirius red for collagen stanning. Scale: 200 µm.

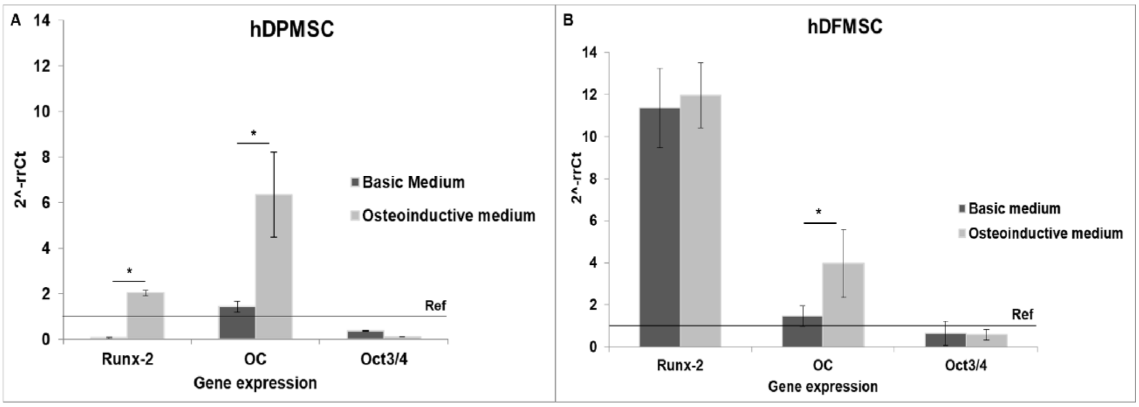

Supplementary Figure 5: Quantitative real-time polymerase chain reaction (qPCR) for osteogenic gene expression (Runx-2 and osteocalcin - OC) and mesenchymal gene expression (Oct3/4) of the human pulp cells (hDPMSC) and follicle cells (hDFMSC) cultured in basic vs. osteoinductive medium for 21 days. Quantification of data was performed using the ΔΔCt method using GAPDH gene expression as an endogenous reference. Results were normalized to the undifferentiated dental cells (P6) average results, and are represented as fold change.

Before performing the *in vivo* cell-loaded scaffold subcutaneous implantation, the scaffolds were seeded with hDPMSC and hDFMSC and cultured with basic and osteoinductive medium for 7 days under dynamic conditions. The samples were evaluated by DNA quantification, ALP activity, osteogenic gene expression (Runx-2, OC and BMP-2) and histological sections were stained with H&E. The results showed no statistical different between the basic and osteoinductive medium (Supp. Fig. 6). The histology sections showed that after 7 days under dynamic conditions both dental MSCs showed higher cellular presence on the scaffolds surface.

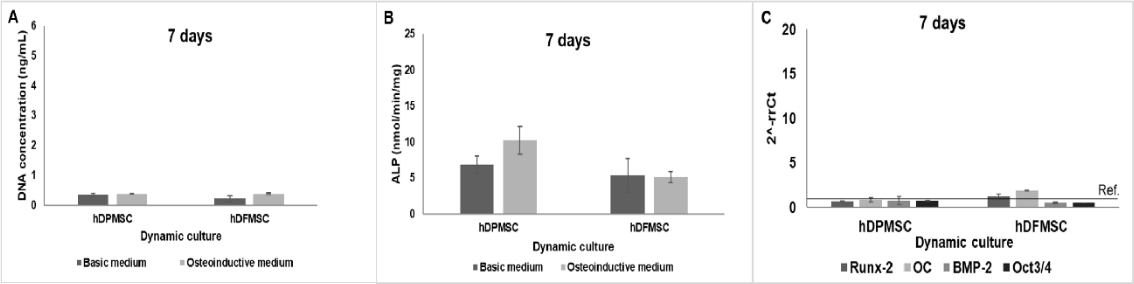

Supplementary figure 6: (A) DNA concentration of hDPMSC and hDFMSC cultured within Coll-nanoHA/OPS for 7 days under basic vs. osteoinductive medium at dynamic conditions. (B) ALP activity on human dental follicle and pulp MSCs cultured within Coll-nanoHA/OPS for 7 days under dynamic conditions. (C) Quantitative real-time

polymerase chain reaction (qPCR) for osteogenic genes (Runx-2, Osteocalcin and BMP-2) and mesenchymal gene (Oct3/4) for dental pulp and follicle MSCs cultured within the scaffold for 7 days with osteinductive medium under dynamic conditions. Quantitative data were calculated by the ΔΔCt method using GAPDH gene expression as an endogenous reference. Results were normalized to the undifferentiated cells (passage 6) average results, and are represented as fold change.

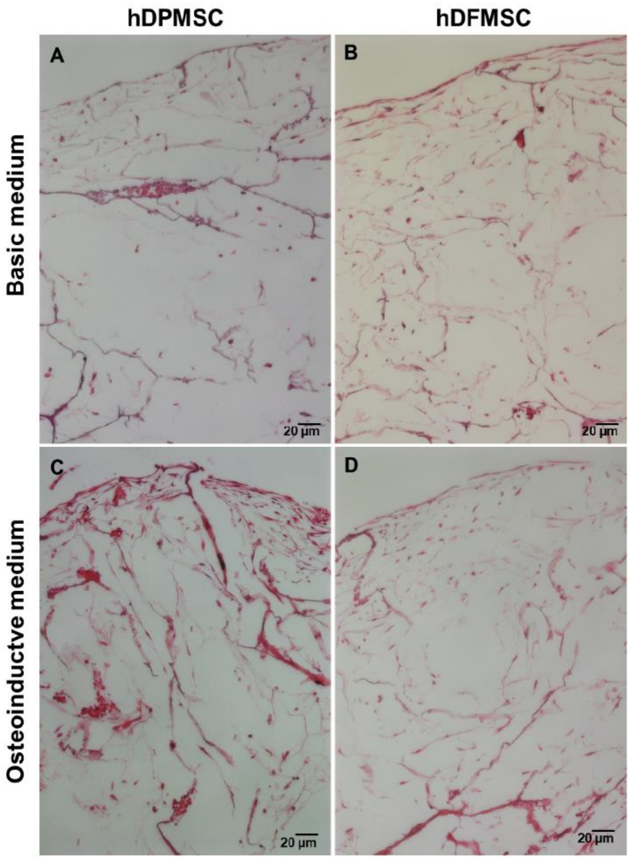

Supplementary figure 7: Optical microscopy images of dental MSCs (hDPMSC: A and C and hDFMSC: B and D) within Collagen-nanoHA/OPS scaffolds under dynamic conditions with basic or osteoinductive culture medium for 7 days. Slides were stained by H&E. Scale: 20 µm.
